# Differences in and Factors Related to Willingness to Provide Care to Patients with Suspected or Confirmed COVID-19 in Long-Term Care Facilities

**DOI:** 10.3390/ijerph192013461

**Published:** 2022-10-18

**Authors:** Jia-Rong Yu, Lan-Ping Lin, Jin-Ding Lin

**Affiliations:** 1 Institute of Long-Term Care, MacKay Medical College, New Taipei City 252, Taiwan; 2 Ren-Ai Senior Citizens’ Home, New Taipei City Government, New Taipei City 207, Taiwan

**Keywords:** coronavirus disease (COVID-19), patients with suspected or confirmed COVID-19, willingness to provide care, trade-off, long-term care, long-term care facilities (LTCF)

## Abstract

The coronavirus disease 2019 (COVID-19) pandemic has exerted tremendous effects on the residents of and caregivers at long-term care facilities (LTCF). The combination of a vulnerable, aged population, staffing shortages, and inadequate resources in LTCF will cause a great negative impact in these sectors. Addressing the caregiver’s lack of interest in providing care for patients with COVID-19 is a great challenge for institutional managers. The primary objective of this study was to analyze the factors related to the willingness of personnel at LTCF to provide care to patients with COVID-19. This was a cross-sectional study in which personnel from 10 LTCF were recruited as participants through convenience sampling and completed structured questionnaires. A total of 500 questionnaires were distributed and 385 valid questionnaires were recovered, posting a response rate of 77%. A statistical analysis was performed using SPSS 22.0. The results of the survey revealed that only 30% of the participants were willing to provide care to patients with COVID-19; 23% more of the participants were willing to provide such care if their institutions provided sufficient PPE. Regarding other conditions, 31.5% and 76% of the participants expressed that they would be willing to provide such care if their compensation were increased and working hours were reduced. In the univariate analysis, the willingness of participants with different characteristics (job categories, years of holding a professional certificate, job location type, monthly income, experience with caring for patients with confirmed COVID-19, and completion of training related to communicable disease control) varied significantly (*p* < 0.05). Furthermore, in the logistic regression analysis, several demographic and professional characteristics (education level, job category, number of patients served daily, and monthly income) were significantly correlated with willingness to provide care to patients with COVID-19 (*p* < 0.05). On the basis of these findings, the LTCF should securitize the associated factors of care wiliness in personnel to eliminate the difference of the willingness to provide care to patients with suspected or confirmed COVID-19.

## 1. Introduction

The coronavirus disease 2019 (COVID-19) pandemic had a great negative impact on long-term care facilities (LTCF). Residents in LTCF are at high risk due to the COVID-19 pandemic and its severe outcomes make recognition of typical COVID-19 symptoms challenging [1]. No effective treatment is currently available for COVID-19, and COVID-19 is highly infectious and potentially deadly. The COVID-19 related deaths in LTCF residents represent 30–60% of all COVID-19 deaths in many European countries [2]. Challenges abound for fully understanding the burden of COVID-19 in LTCF, including differences in nomenclature, data collection systems, cultural differences, varied social welfare models, and under-resourcing of these sectors [3]. Therefore, LTCF should consider implementing a daily surveillance routine for their residents to detect COVID-19 infections, enabling an appropriate implementation of infection prevention measures, and limiting the size of outbreaks in residents and staff [4]. 

Compared with other occupations, personnel at LTCF, who are often in long-term close contact with residents, are at higher risk of exposure to COVID-19, and many such personnel have insufficient knowledge regarding how to care for residents with suspected or confirmed COVID-19. Sun et al. [5] indicated that the positive and negative emotions of frontline nurses interweaved and coexisted during a COVID-19 epidemic outbreak. Numerous studies have noted that when nursing personnel are required to provide care to patients with suspected or confirmed COVID-19, they endure great psychological stress and negative emotions, and are subject to a greater workload and greater work stress. Zhang et al. [6] surveyed frontline nursing personnel in China to investigate how the COVID-19 pandemic affected their mental health. They reported that the workload and work stress may result in anxiety, insomnia, fatigue, depression, fear of infection, ambivalence, emotional exhaustion, posttraumatic stress disorder, and other mental disorders. One qualitative study in Taiwan also has confirmed that COVID-19-related stress and psychological distress occurred among Taiwanese nursing personnel [7]. White et al. [8] investigated the experiences of 152 frontline workers (including direct service workers and administrative workers) at US nursing homes (across 32 states) during the COVID-19 pandemic. The workers reported experiencing burnout due to increased workload, labor shortages, and the need to care for residents facing isolation, illness, and death. These challenges place a greater burden on an already tight and vulnerable workforce and can lead to long-term burnout, turnover, and labor shortages.

Numerous quantitative studies have examined healthcare professionals’ knowledge of and attitude toward COVID-19 prevention and treatment. One study, in Egypt, examined 407 frontline caregivers’ knowledge of, attitude toward, and views on COVID-19; 83.1% of the participants responded that they were afraid of contracting COVID-19, and 89.2% indicated they were especially susceptible to contracting COVID-19. The problems they faced included a lack of personal protective equipment (PPE), fear of spreading the disease to family members, and social stigma [9]. Another study of 10 hospitals in Henan, China evaluated 1357 frontline health caregivers’ knowledge of, attitude toward, and ability to provide clinical nursing services to patients with COVID-19 [6]. They found that 89% of the participants had a sufficient understanding of COVID-19, over 85% were concerned about contracting COVID-19, and 89.7% followed recommended COVID-19-related practices. However, more than 85% still feared self-infection with the virus. A US study conducted in 2021 evaluated the attention of senior nursing students and interns to patients with COVID-19 and their willingness to treat such patients [10]; the results showed that the students and interns were more willing to care for patients with COVID-19 when they possessed more relevant knowledge, and that possessing overall professional knowledge, the professional knowledge required for their roles, and awareness of the importance of their roles exhibited positive effects on their willingness to care for said patients during the pandemic. Sufficient PPE and health protection measures for individuals as well as their family and friends were also determined to positively affect the participants’ willingness to treat patients with COVID-19.

According to the provisions of Article 3 of the Long-term Care Services Act in Taiwan, long-term care is defined as “the living support, assistance, social participation, care and relevant health-care services in accordance with the needs of any individual whose mental or physical incapacity has lasted or is expected to last for six months or longer, or the needs of such an individual’s care provider” [11]. The service targets include not only patients but also caregivers. In summary, the decision of whether to provide care to patients with COVID-19 is based on healthcare personnel’s attitude toward the patients as well as the personnel’s motivations, values, and cognition including their personal motivation, self-commitment, responses to others’ expectations, civic and social responsibilities, social recognition and compensation, personal growth, and interpersonal relationships. Willingness refers to the intention to engage in a certain job or role; in the case of caregivers, it may refer to an individual’s intention to devote considerable energy and emotional labor to their work, contribute their skills, and share responsibilities to provide practical help, companionship, and professional care [12]. The factors related to the willingness of frontline personnel to provide care to patients with COVID-19 identified in the aforementioned studies can be categorized as personal motivations and work incentives. However, high-quality evidence of the effectiveness in protecting long-term care facilities from COVID-19 was limited [13], and there are no studies conducted in Taiwan that have evaluated the willingness of personnel at LTCF to provide care to patients with COVID-19. To address this gap, the present study explored the factors related to willingness of personnel at LTCF in Taiwan to provide care to patients with COVID-19. The main hypotheses in this study propose that there is a significant difference between the personnel’s demographic, working, and epidemic disease training/serving experience variables and the willingness to care for COVID-19 cases. The results of this study may serve a reference for the development of relevant measures and intervention to make personnel at LTCF more willing to care for patients with COVID-19 in the future.

## 2. Research Methods

This study employed a cross-sectional design, and participants were recruited through convenience sampling. The research plan was approved by the Institutional Review Board of Taipei Mackay Memorial Hospital (No. 21MMHIS379e). According to the Taiwan Ministry of Health and Welfare, as of June 2021, there were 1078 LTCF and 21,944 professional primary caregivers (including directors, supervisors, nurses, nursing aide, social workers, and other personnel) [14]. The present study involved a survey of members of this workforce. The required sample size was calculated using the online version of Raosoft software [15]; the results indicated that 385 valid samples were required to obtain a 95% confidence interval while accounting for a sampling error of 5% and effect size of 0.2. All the personnel from 10 LTCF were invited to participate in this survey (excluding caregivers of foreign nationality). A total of 500 questionnaires were distributed and 385 valid questionnaires were recovered, posting a response rate of 77%.

A self-administered structured questionnaire, designed according to the research objective, research framework, and the relevant literature, was used for data collection. The questionnaire includes two main parts—demographic characteristics (social economic status, job experience, infectious disease training, and service experience) and a willingness-to-care scale (4 domains). The participants were required to answer whether they were willing or unwilling to provide care (or “no comment”) and to explain their reasoning. Additionally, they were required to expound which trade-off conditions they were willing to accept in exchange for providing care to patients with suspected or confirmed COVID-19. The willingness of the participants to provide care to patients with COVID-19 was motivated by self-determination factors including personal motivation, self-commitment, responding to the expectations of others, civic responsibilities, social care, social recognition, and work incentives and compensation. These factors were summarized as personal motivation and work-incentive-based factors. The face validity of the questionnaire was evaluated by 5 experts whose professional fields include social work (1), public health (2), social welfare (1), and nursing expert (1). The average content validity index was 0.84, indicating that the questionnaire had excellent validity. Regarding the reliability of the questionnaire, the Cronbach’s α coefficient of the questionnaire was 0.95. The data collection procedure of this study began with a description of the purpose, content, subjects, and related procedures of the study, and consent was obtained from each LTCF. The study was conducted by researchers in person at each LTCF and data was collected after the subjects signed a consent form. After the questionnaires were completed by the participants and collected by the researchers, the data were archived using Microsoft Excel, and a descriptive and inferential statistical analysis was performed using SPSS 22.0.

## 3. Results

### 3.1. Willingness to Provide Care to Patients with COVID-19 in Different Scenarios

The total number of participants in this study was 385. In terms of gender distribution, 307 (79.7%) of the participants were female. The age group was mostly 50–59 years old, accounting for 122 (31.7%). In terms of job characteristics, the majority of the job categories were caregivers, accounting for 184 (47.8%), nurses (25.7%), social workers (8.3%), managers (5.2%), and administrative officials (9.9%). Among the surveyed personnel, 119 (30.9%) and 156 (40.4%) were willing and unwilling, respectively, to provide care to suspected or confirmed patients with COVID-19, and 110 (28.6%) had no comment (Table 1). Among those who were willing to provide such care, 87 (33.2%) and 76 (29%) cited that the primary reason for their response was their sense of professional responsibility and the opportunity for personal learning and growth, respectively. Among those who were unwilling to provide such care, 98 (29.4%) and 82 (24.6%) cited that the primary reason for their response was their lack of expertise on providing such care and their reluctance to risk contracting COVID-19, respectively.

**Figure 1:**
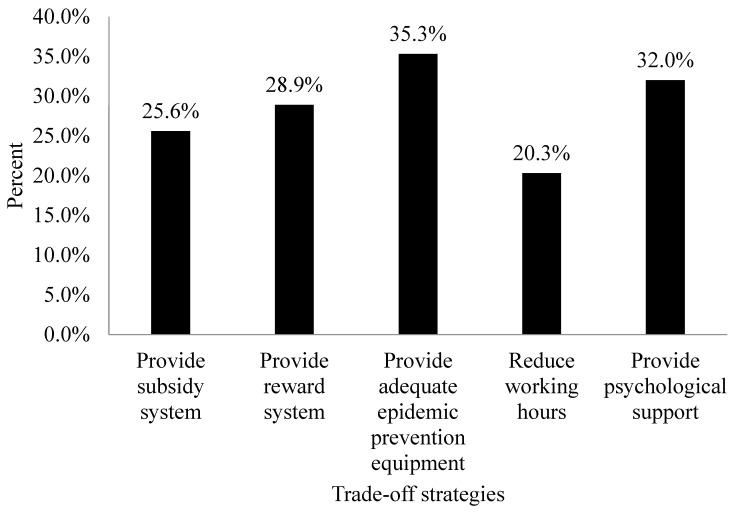
Willingness to provide care to patients with COVID-19 under different trade-off conditions (*n* = 266).

Regarding the effects of various work incentives on the participants’ willingness to provide care to patients with COVID-19, 94 (35.3%), 85 (32%), 77 (28.9%), 68 (25.6%), and 54 (20.3%) of the participants were willing to provide such care if their institutions were to offer sufficient PPE, mental health support programs, incentive systems, subsidy systems, and reductions in working hours, respectively (Figure 1).

### 3.2. Univariate Analysis of Factors Related to Willingness to Provide Care

The effects of demographic variables on the participants’ willingness to provide care to patients with COVID-19 were analyzed. Willingness to provide care to patients with COVID-19 was set as the dependent variable (with responses indicating unwillingness and refusal to comment both treated as expressions of unwillingness); and the basic demographic variables, which combined job characteristics and certain variables, were the independent variables. A univariate analysis was then conducted. The participants’ ages were divided into four groups (20–35, 36–51, 52–64, and ≥65 years). Their education levels were classified as junior high school or below, senior high school, and junior college or above; their marital statuses were categorized into (1) unmarried, divorced or widowed and (2) married; and their self-rated health statuses were classified as healthy, normal, or unhealthy. Their job titles were categorized into four groups (caregiver, nurse, social worker, and others). The durations for which they had held professional certificates were divided into three groups (<1 year, 1–5 years, and >5 years), and the numbers of patients they served per day were divided into four groups (1–10 people, 11–15 people, ≥16 people, and uncountable [for those who participated in indirect service]). Regarding the participants’ monthly income, the participants’ monthly income was ultimately divided into three groups (<USD 1000, USD 1000–1500, and >USD 1500).

In the univariate analysis of the participants’ background information and willingness to provide care to patients with COVID-19, the dependent variable (willingness to provide care to patients with COVID-19) and the independent variables (basic demographic variables, job characteristics, and COVID-19 treatment and control experience) were all categorical variables.

A chi-squared test was used to analyze the collected data. The results of the analysis (Table 2) indicated that the relationships between demographic variables and the participants’ willingness to provide care to patients with COVID-19 were nonsignificant. Regarding job characteristics (Table 3), job category (*p* = 0.001), years of holding a professional certificate (*p* = 0.006), employment at a LTCF (*p* = 0.001), and monthly income (*p* < 0.001) were significantly associated with willingness to provide care to patients with COVID-19; the remaining variables were not. Regarding job category, 42 (64.6%) of the nurses were willing to provide care to patients with COVID-19; this proportion was greater than that of the participants with other job titles. In addition, 82 (50.3%) of the participants who had held a professional certificate for >5 years were more willing to provide such care than those who held a professional certificate for different durations.

Concerning COVID-19 treatment and control experience (Table 4), completion of training related to communicable disease control (*p* = 0.003) was significantly associated with willingness to provide care to patients with COVID-19; the remaining variables were not. Of the participants who had received training related to communicable disease control, 85 (50.3%) were willing to provide care to patients with COVID-19; this proportion was greater than that of the participants who had not received relevant training.

### 3.3. Logistic Regression Analysis of Factors Related to Willingness to Provide Care

The results of the logistic regression analysis are presented in Table 5. According to the research framework, the participants’ background information (demographic variables, job characteristics, COVID-19 treatment, and control experience) was substituted into the logistic regression model, and three models were established. In Model 1, which included only the participants’ basic demographic variables, an age of 51–64 years (OR = 3.42; 95% CI = 1.09–10.78; *p* = 0.036) was significantly associated with willingness to provide care to patients with COVID-19. In Model 2, which included both basic demographic variables and job characteristics, an education level of junior college and above (OR = 0.39; 95% CI = 0.16–0.95; *p* = 0.038), employment as a nurse (OR = 3.39; 95% CI = 1.21–9.50; *p* = 0.021), and a monthly income of >USD 1500 (OR = 5.22; 95% CI = 1.56–17.47; *p* = 0.007) were significantly associated with willingness to provide care to patients with COVID-19. In Model 3, which included basic demographic variables, job characteristics, and COVID-19 treatment and control experience, an education level of junior college and above (OR = 0.37; 95% CI = 0.14–0.89; *p* = 0.046), employment as a nurse (OR = 4.03; 95% CI = 1.38–11.77; *p* = 0.011), participation in indirect service (OR = 0.30; 95% CI = 0.11–0.83; *p* = 0.002), a monthly income of >USD 1500 (OR = 5.26; 95% CI = 1.48–18.61; *p* = 0.010), and experience providing care to patients with confirmed COVID-19 (OR = 9.93; 95% CI = 1.11–89.07; *p* = 0.040) were significantly associated with willingness to provide care to suspected or confirmed patients with COVID-19 than their counterparts.

## 4. Discussion

### 4.1. Differences in Personnel’s Willingness to Provide Care to Patients with COVID-19

In response to COVID-19, Taiwan established the Central Command Center for COVID-19 on 20 January 2020 [16]. In view of the continued expansion of the epidemic in various countries, the command center coordinates the resources and manpower of various ministries, strengthens the coordination between the command center and county and municipal governments, and actively plans and implements various response strategies in order to reduce the risk of introduction and community transmission of the epidemic and to ensure public health. In terms of LTCF, the Ministry of Health and Welfare’s Center for Disease Control have established guidelines for LTCF to reduce the risk of transmission of the novel coronavirus in institutions. Accordingly, the staff of LTCF includes caregivers, social workers, care attendants, administrative staff, nursing staff, and other professional staff, etc. They are important players in the implementation of the above guidelines related to infection control in LTCF.

In the present study, the participants who were unwilling to provide care to patients with COVID-19 outnumbered those who were willing to provide such care. Among those who were willing to provide such care, the most frequently cited reason for their willingness was their sense of professional responsibility. Previous studies have reported that healthcare personnel charged with caring for patients with COVID-19 were under great physical and mental pressure during the pandemic [17,18]. Providing mental health aid should thus be an essential part of services for healthcare providers during the pandemic [18]. They exhibited tremendous strength and resilience, drawing multiple support systems and self-adjustment skills to manage their stress because they were aware of the need to be strong and focus on their responsibilities to save lives [19]. The most frequently cited reason why many of the participants were unwilling to provide care to patients with COVID-19 was a lack of relevant expertise. Among the participants who were unwilling to provide care to patients with COVID-19, social workers accounted for the greatest proportion, followed by participants with other job titles (administrators, supervisors, managers, dietitians, physiotherapists, occupational therapists, pharmacists, drivers, janitors, security guards, and accountants). Because social workers and other personnel are not frontline direct caregivers, they may lack sufficient professional healthcare knowledge and therefore feel incapable of providing care to patients with COVID-19. This finding is consistent with the findings of a foreign study that explored the challenges faced by frontline social workers during the COVID-19 pandemic in which psychological support workers help frontline workers manage their anxiety and fear of COVID-19 rooted in their inability to treat cases and experience of seeing other frontline workers lose their lives [20]. Therefore, providing additional relevant professional training courses to non-frontline care personnel is crucial to increasing their willingness to provide care to patients with COVID-19.

### 4.2. Experience with Communicable Disease Control on Willingness to Provide Care to Patients with COVID-19

In view of the challenges, due to the lack of information and competencies in infection prevention and control at LTCF [21], evidence suggests the need to develop a plan that provides support to those in need of care and their families, and that includes LTCF. According to the present results of the univariate analysis, among the various types of personnel at LTCF, nurses are the most willing to provide care to patients with COVID-19. Nyashanu et al. [20] explored the challenges faced by frontline healthcare professionals and social workers during the COVID-19 pandemic and discovered that many nurses believe that they are responsible for protecting the individuals they are tasked with providing care to because of the concept of social responsibility. In the present study, the longer the durations for which the participants had held a professional certificate were, the more willing they were to provide care to patients with COVID-19. This result is consistent with those of the aforementioned study on stress, psychological distress, and stress-relief strategies among Taiwanese nursing personnel during the COVID-19 pandemic, in which stress-relief strategies were determined to be positively correlated with years of employment. Nurses with more years of experience were more likely to identify effective stress relief methods they could use to cope with the COVID-19 pandemic and could therefore continue to provide care to patients [7]. In addition, senior nurses tend to have more knowledge and expertise and are therefore better equipped to deal with difficult situations and less prone to burnout [22]. The nursing role for care stewardship within LTCF is crucial, and the nursing workforce truly engaged in working with interdisciplinary colleagues and focusing on care planning that includes medication management to improve the health status of residents in LTCF [23].

Regarding COVID-19 treatment and control experience, the participants who had previously cared for patients with confirmed COVID-19 were more likely to be willing to care for patients with suspected or confirmed COVID-19. These findings are consistent with those of Shi et al. [24], who studied knowledge of and attitude toward COVID-19 among healthcare professionals at hospitals in China. Moreover, Feng et al. [7] reported that nurses who have experience with caring for patients with communicable diseases had higher total stress relief scores, which is consistent with the finding of the present study that personnel who had received training related to communicable disease control were more willing to provide care to patients with COVID-19. Likewise, Alshutwi [10] reported that nursing students were more willing to provide care to patients with COVID-19 if they had acquired more information about COVID-19.

### 4.3. Effects of Various Work Incentives on Willingness to Provide Care to Patients with COVID-19 

Workers facing the highly infectious COVID-19 novel disease are affected by various internal and external factors, such as knowledge of the disease, psychological stress, social and family factors, etc., which may affect the willingness to take care of the case [25,26]. According to Quigley et al. [27], some nursing home supervisors claimed that insufficient PPE and labor shortages were the largest and most common problems facing LTCF during the COVID-19 pandemic. The results of the present study demonstrated that LTCF can increase the number of their personnel willing to provide care to patients with COVID-19 by providing sufficient PPE, especially given that PPE has remained in extremely high demand as healthcare professionals and the public have continued to fight against the COVID-19 pandemic [28]. During the COVID-19 pandemic, frontline workers employed in workplaces without sufficient PPE were at risk of contracting COVID-19, creating fear and anxiety among such workers, especially those tasked with providing direct care to patients with COVID-19 [20]. 

Moreover, in the present study, an increase in the month subsidy in compensation was determined to considerably improve the participants’ willingness to provide care to patients with COVID-19. Fisher et al. [29] demonstrated that though the implementation of specific measures to support and protect employees (including providing hazard allowances, other employee benefits, child allowances, and paid leave), some of the negative effects of the COVID-19 pandemic on personnel could be mitigated. Some healthcare institutions in Taiwan have provided subsidies and established incentive systems to facilitate the provision of care to patients with confirmed COVID-19. For example, Far Eastern Memorial Hospital-recruited individuals under the age of 65 years with confirmed cases of COVID-19, who had been released from quarantine and had received three doses of COVID-19 vaccines, were to provide care to patients with suspected or confirmed COVID-19. The recruited individuals received USD 250 for 12 h of labor or USD 166 for 8 h of labor and were required to feed, turn over, and change the diapers of the patients as necessary. The vacancies were filled within a few days [30]. In addition to compensation, the results of the present study indicated that a reduction of 10–39 working hours per month could increase personnel’s willingness to provide care to patients with COVID-19. Because of the shortage of and increased demand for professionals willing and able to care for patients with confirmed COVID-19 during the pandemic, hospitals relied on healthcare professionals volunteering to work extended hours and support from nonclinical personnel. Some hospitals even hired contract personnel or forced employees to extend their working hours [27]. 

The COVID-19 pandemic provoked a generalized climate of wariness and uncertainty for health professionals due to a range of causes such as the virus rapid spread, the severity of symptoms and the lack of knowledge of the disease, and deaths [31]. During the pandemic, personnel at healthcare institutions often worked overtime, which increased the physical burden of their work. Therefore, reducing the working hours of such personnel could help them rest and, in turn, motivate them to continue working. Finally, the results of the present study indicated that the provision of mental health support programs can increase willingness of personnel to provide care to patients with COVID-19, which is consistent with the results of a study conducted in China [32]. The local government of Wuhan, China implemented a mental health treatment program through which healthcare teams provide psychotherapy (including telephone guidance) to relevant personnel. These services helped relive the stress of hundreds of healthcare personnel who received treatment through the program and enabled them to continue to provide care to patients with confirmed COVID-19.

Ignatius Brereton [33] suggests the use of the theory of organizational incentives, which engages with value-based models as a starting point to consider organizational-level incentive possibilities or consider an organizational-level pay for performance model, a time-bound incentive structure, and investor-specific incentives to long-term care during the COVID-19 pandemic. In this study, many incentives, such as providing appropriate care design for healthcare personnel, subsidized or free accommodations, subsidies for intensive caregiving, one-person/one-room accommodations, government subsidies and bonuses, and PPE can increase the willingness of personnel to provide care to patients with COVID-19. The results are consistent with those reported by Martin [34], who studied the ability and willingness to work for nursing personnel during a pandemic and determined that 90.1% of nursing personnel would be willing to work during a pandemic and identified the main reasons for unwillingness to work as shortages of PPE, fear of putting oneself or one’s family members at risk, the need to take care of family members, and lack of access to transportation. In addition, Feng et al. [7] explored stress, psychological distress, and stress-relief strategies among Taiwanese nursing personnel during the COVID-19 pandemic and discovered that many nurses were worried about infecting their relatives and friends as well as about being separated from their family members after being infected. The nurses with school-aged children experienced even greater stress, especially because of their concerns about their family members being discriminated against or infected during the pandemic. Therefore, when caring for patients with COVID-19, caregivers must consider not only themselves but also their families.

### 4.4. Relationships between Demographic Variables and Willingness to Provide Care to Patients with COVID-19

Kanios and Bocheńska-Brandt [35] indicated that workers at LTCF are at risk of occupational burnout, and that statistically significant differences existed depending on their personality traits in all the inventories analyzed: emotional exhaustion, depersonalization, and personal accomplishment. Care for COVID-19 suspected or confirmed patients in LTCF is based on personal motivation, values, perceptions of the case and psychological intentions toward the case, and whether to provide services to the case. In this study, some factors that were significantly related to the willingness of personnel at LTCF to provide care to patients with COVID-19 were identified. The participants with an education level of senior high school or junior college or above were less willing to provide care to patients with COVID-19 than those with an elementary education level. The nurses were more willing to provide such care than the caregivers, and participants who participated in indirect service were less willing to provide care to patients with COVID-19 than participants who reported providing care to 1–10 people per day were. The participants with a higher monthly income were more willing to provide such care than were those with less monthly income, indicating that sufficient compensation is crucial to increasing the willingness of personnel to provide care to patients with COVID-19. In summary, several factors can increase or decrease the willingness and ability of frontline workers to provide care to patients during a pandemic. Awareness of these factors can help LTCF prepare for emergencies and prevent turnover.

### 4.5. Analysis of Factors Related to Personnel’s Willingness to Provide Care to Patients with COVID-19

The staff of LTCF are essential to the care and prevention of major infectious diseases and therefore need to have a good health awareness. In this study, the relationship between the participants’ completion of training related to communicable disease control and their willingness to provide care to patients with COVID-19 was statistically nonsignificant. This finding differs from that of Alshutwi [10], who indicated that nursing students’ knowledge of and attention to the treatment of COVID-19 can predict their willingness to provide care to patients with COVID-19, which may be because of the willingness of personnel at LTCF to provide care to patients with COVID-19 being affected by additional factors. Aoyagi et al. [36] identified various factors, such as male gender, employment as a nurse or physician, fulltime employment, clinical knowledge and risk assessment skills, confidence in skills, and communicable disease control training, that were correlated with caregivers’ willingness to work during the COVID-19 pandemic. According to Martin [34], predictors of personnel’s willingness to care for patients with communicable diseases include the provision of sufficient PPE and measures to protect the health of personnel as well as their family and friends. In a study by Feng et al. [7], many Taiwanese nursing personnel reported being worried about infecting their relatives and friends and about being separated from their families after being infected. Those with school-aged children were even more stressed, mainly because they feared that their family members would be infected or discriminated against during the pandemic. In the present study, apart from the most commonly-cited reason for being unwilling to provide care to patients with COVID-19 (i.e., lack of relevant expertise), many participants cite their fear of being infected or of infecting their family members. In addition to personal factors, familial factors also affect willingness of personnel at LTCF to provide care to patients with COVID-19. Therefore, only by understanding the relevance of COVID-19 health awareness and case care willingness of staff in LTCF, and by providing adequate support or reducing these factors to staff, can the crisis of the epidemic be successfully overcome and the impact of the epidemic on staff be minimized.

The literature review, research design, questionnaire distribution, and statistical analysis involved in the present study were objective and discreet. However, this study still has some limitations. The inferences that can be made on the basis of data collected through convenience sampling are limited, and ongoing changes associated with the pandemic have limited the timeliness of research results. In addition, the questionnaire survey coincided with the increasing peak of the pandemic, and the participants were therefore under particularly large work stress. Another limitation is the absence of evaluation of psychological characteristics that can play a role on the willingness to provide care to patients with suspected or confirmed COVID-19 as burnout of the participants. Finally, because this study was cross-sectional, no causal inferences could be firmly drawn on the basis of the analyses.

## 5. Conclusions

This study explored the profile and factors related to willingness of personnel at LTCF in Taiwan to provide care to suspected or confirmed patients with COVID-19. The findings illustrated that only less than one third were willing to provide care to suspected or confirmed patients with COVID-19. Among those who were willing to provide such care, the most frequently cited reason for their willingness was their sense of professional responsibility. On the basis of the findings, this study suggests that LTCF should securitize the associated factors of care willingness in personnel to eliminate the difference of the willingness to provide care to patients with suspected or confirmed COVID-19 to improve and maintain the health of the elderly. 

## Figures and Tables

**Table 1:** Distribution of participants’ willingness to provide care to patients with COVID-19 (*n* = 385).

Item	Willing to Provide Care	Unwilling to Provide Care	No Comment
	*n* (%)	*n* (%)	*n* (%)
Willingness to provide care to patients with suspected or confirmed COVID-19	119 (30.9)	156 (40.5)	110 (28.6)
1. Reasons for willingness to provide care (multiple choice)			
1.1. Responsibility as a professional	87 (33.2)		
1.2. Opportunities for personal learning and growth	76 (29.0)		
1.3. Performance of job duties	70 (26.7)		
1.4. Previous experience with caring for patients with COVID-19	13 (5.0)		
1.5. Completion of training or courses related to caring for patients with COVID-19	13 (5.0)		
1.6. Others	3 (1.1)		
2. Reasons for unwillingness to provide care (multiple choice)			
2.1. Not considering taking care of patients with suspected or confirmed COVID-19 to be an institutional responsibility		31 (9.3)	
2.2. Lack of relevant expertise		98 (29.4)	
2.3. Reluctance to risk exposure to the virus		82 (24.6)	
2.4. Insufficient care equipment and human resources at the institution		65 (19.5)	
2.5. Lack of proper PPE		37 (11.1)	
2.6. Others		20 (6.0)	
3. Trade-off conditions (incentives offered by institutions; *n* = 266)			
3.1. Subsidy systems (e.g., subsidies related to contracting COVID-19 at work)	68 (25.5)	93 (35.0)	105 (39.5)
3.2. Incentive systems (e.g., epidemic prevention bonuses and care bonuses)	77 (28.9)	94 (35.3)	95 (35.7)
3.3. Sufficient PPE	94 (35.4)	90 (33.8)	82 (30.8)
3.4. Reductions in working hours	54 (20.3)	126 (47.4)	86 (32.3)
3.5. Mental health support programs	85 (32.0)	101 (38.0)	80 (30.0)

**Table 2:** Univariate analysis of demographic variables and willingness to provide care to patients with COVID-19 (*n* = 275).

Variable	Willingness to Provide Care		χ²	*p*
	Willing to Provide Care (%)	Unwilling to Provide Care (%)		
Gender			1.50	0.221
Male	21 (36.2)	37 (63.8)		
Female	98 (45.2)	119 (54.8)		
Age (years)			5.83	0.121
20–35	6 (25.0)	18 (75)		
36–50	41 (41.4)	58 (58.6)		
51–64	53 (49.1)	55 (50.9)		
≥65	19 (43.2)	25 (56.8)		
Education level			4.89	0.180
Junior high school or below	37 (59.7)	25 (40.3)		
Senior high school	61 (73.5)	22 (26.5)		
Junior college or above	168 (70.0)	72 (30.0)		
Marital status			0.29	0.589
Unmarried, divorced, or widowed	39 (41.1)	56 (58.9)		
Married	80 (44.4)	100 (55.6)		
Religious belief			2.98	0.394
None	43 (40.6)	63 (59.4)		
Buddhist	34 (41.0)	49 (59.0)		
Christian	13 (40.6)	19 (59.4)		
Others *	29 (53.7)	25 (46.3)		
Breadwinner			0.078	0.780
Yes	60 (44.1)	76 (55.9)		
No	59 (42.4)	80 (57.6)		
Self-rated health				
Healthy	70 (43.2)	92 (56.8)	0.242	0.886
Normal	43 (42.6)	58 (57.4)		
Unhealthy	6 (50.0)	6 (50.0)		

* Yiguandao + Catholic.

**Table 3:** Univariate analysis of job characteristics and willingness to provide care for patients to COVID-19 (*n* = 275; continued).

Variable	Willingness to Provide Care		χ²	*p*
	Willing to Provide Care (%)	Unwilling to Provide Care (%)		
Total years of employment at current institution			0.60	0.741
1–10	86 (42.0)	119 (58.0)		
11–20	22 (47.8)	24 (52.2)		
>20	11 (45.8)	13 (54.2)		
Number of patients served daily			2.17	0.537
1–10	37 (43.5)	48 (56.5)		
11–15	11 (50.0)	11 (50.0)		
≥16	44 (48.4)	47 (51.6)		
Uncountable	27 (35.1)	50 (64.9)		
Service hours per month			1.06	0.787
40–79	9 (34.6)	17 (65.4)		
80–119	6 (40.0)	9 (60.0)		
120–159	14 (46.7)	16 (53.3)		
≥160	90 (44.1)	114 (55.9)		
Monthly income (USD)			15.84	<0.001
<1000	10 (22.2)	35 (77.8)		
1000–1500	75 (42.9)	100 (57.1)		
>1500	34 (61.8)	21 (38.2)		

**Table 4:** Univariate analysis of relevant epidemic prevention (treatment and control) experience and willingness to provide care to patients with COVID-19 (*n* = 275).

Variable	Willingness to Provide Care		χ²	*p*
	Willing to Provide Care (%)	Unwilling to Provide Care (%)		
Previous experience with caring for patients with suspected COVID-19			0.31	0.576
Yes	8 (50.0)	8 (50.0)		
No	111 (42.9)	148 (57.1)		
Previous experience with caring for patients with confirmed COVID-19			3.54	0.060
Yes	10 (41.2)	5 (58.8)		
No	109 (41.9)	151 (58.1)		
Vaccination			2.64	0.104
Yes	117 (42.9)	156 (57.1)		
No	2 (100.0)	0 (0)		
Completion of training related to communicable disease control			8.81	0.003
Yes	85 (50.3)	84 (49.7)		
No	34 (32.1)	72 (67.9)		

**Table 5:** Logistic regression analysis of willingness to provide care to patients with COVID-19 (*n* = 275) *.

Variable	Model 1			Model 2			Model 3		
	OR	95% CI	*p*	OR	95% CI	*p*	OR	95% CI	*p*
Age (≤35 years)	1.00			1.00			1.00		
36–50 years	2.30	0.78–6.81	0.131	1.96	0.56–6.80	0.290	3.16	0.76–13.18	0.114
51–64 years	3.42	1.09–10.78	0.036	3.40	0.91–12.68	0.068	5.61	1.25–25.23	0.025
≥65 years	2.25	0.73–6.94	0.157	3.30	0.90–12.02	0.072	5.65	1.29–24.75	0.021
Education level (ref: Junior high school or below)	1.00			1.00			1.00		
Senior high school	0.49	0.22–1.09	0.079	0.39	0.16–0.95	0.038	0.37	0.15–0.90	0.028
Junior college and above	0.80	0.40–1.61	0.536	0.44	0.17–1.14	0.092	0.37	0.14–0.89	0.046
Job category (ref: Caregiver)				1.00			1.00		
Nurse				3.39	1.21–9.50	0.021	4.03	1.38–11.77	0.011
Social worker				0.72	0.17–3.06	0.652	1.23	0.27–5.64	0.787
Others **				1.03	0.34–3.07	0.964	1.49	0.47–4.72	0.494
Number of patients served daily (ref: 1–10)				1.00			1.00		
11–15				1.06	0.35–3.24	0.920	0.90	0.28–2.88	0.863
≥16				0.49	0.22–1.11	0.087	0.44	0.19–1.02	0.055
Indirect service				0.41	0.16–1.04	0.061	0.30	0.11–0.83	0.020
Monthly income (ref: <1000 USD)				1.00			1.00		
1000–1500				2.20	0.83–5.83	0.112	2.22	0.81–6.09	0.120
>1500				5.22	1.56–17.47	0.007	5.26	1.48–18.61	0.010
Experience with caring for patients with confirmed COVID-19 (ref: No)							1.00		
Yes							9.93	1.11–89.07	0.040

* Adjusted factors: gender, marital status, religious belief, self-rated health, years of holding a professional certificate, total years of service in long-term care, total years of employment at current institution, service hours per month, experience with caring for patients with suspected COVID-19, vaccination, completion of training related to communicable disease control, and type of institution. ** Administrator, supervisor, manager, dietitian, physiotherapist, occupational therapist, pharmacist, driver, janitor, security guard, and accountant.

## Data Availability

The data that support the findings of this study are available on request from the corresponding author. The data are not publicly available due to their containing information that could com-promise the privacy of research participants.
